# Coffee Intake and Neurocognitive Performance in HIV/HCV Coinfected Patients (ANRS CO13 HEPAVIH)

**DOI:** 10.3390/nu12092532

**Published:** 2020-08-21

**Authors:** Saskia Antwerpes, Camelia Protopopescu, Philippe Morlat, Fabienne Marcellin, Linda Wittkop, Vincent Di Beo, Dominique Salmon-Céron, Philippe Sogni, Laurent Michel, Maria Patrizia Carrieri, the ANRS CO13 HEPAVIH Study Group

**Affiliations:** 1 Aix Marseille Univ, INSERM, IRD, SESSTIM, Sciences Économiques & Sociales de la Santé & Traitement de l’Information Médicale, 13385 Marseille, France; saskia.antwerpes@univ-amu.fr (S.A.); fabienne.marcellin@inserm.fr (F.M.); vincent.di-beo@inserm.fr (V.D.B.); maria-patrizia.carrieri@inserm.fr (M.P.C.); 2 ORS PACA, Observatoire Régional de la Santé Provence-Alpes-Côte d’Azur, 13385 Marseille, France; 3 Service de Médecine Interne et Maladies Infectieuses, CHU de Bordeaux, Université de Bordeaux, 33000 Bordeaux, France; philippe.morlat@chu-bordeaux.fr; 4 ISPED, Inserm, Bordeaux Population Health Research Center, Team MORPH3EUS, UMR 1219, CIC-EC 1401, University of Bordeaux, F-33000 Bordeaux, France; linda.wittkop@u-bordeaux.fr; 5 CHU de Bordeaux, Pole de Sante Publique, F-33000 Bordeaux, France; 6 Université Paris Descartes, 75006 Paris, France; dominique.salmon@aphp.fr (D.S.-C.); philippe.sogni@aphp.fr (P.S.); 7 Service Maladies Infectieuses et Tropicales, AP-HP, Groupe Hospitalier Cochin Hôtel Dieu, 75014 Paris, France; 8 Service d’Hépatologie, Groupe Hospitalier Cochin Hôtel Dieu, Assistance Publique-Hôpitaux de Paris, 75014 Paris, France; 9 UMRS 1018, Paris-Saclay University, 94807 Villejuif, France; laurent.michel@croix-rouge.fr; 10 Centre Pierre Nicole, French Red Cross, 75005 Paris, France

**Keywords:** coffee, hepatitis C, HIV, neurocognitive disorders

## Abstract

Coffee is one of the most consumed beverages worldwide. Previous research has demonstrated its neuroprotective effects in the elderly. People coinfected with human immunodeficiency virus (HIV) and hepatitis C virus (HCV) experience an accelerated aging process and cognitive impairment, which significantly impair quality of life and may affect disease-related dimensions such as treatment adherence. This study aimed to analyse the relationship between regular coffee intake and neurocognitive performance (NCP) in HIV-HCV coinfected people. We used data from 139 coinfected patients who participated in both the ANRS CO13 HEPAVIH cohort and the HEPAVIH-Psy cross-sectional survey. Linear regression models adjusting for potential sociodemographic (age, gender, educational level), clinical (liver disease status, ongoing HCV treatment, HIV viral load, major depressive disorder) and socio-behavioural (cannabis use) correlates of NCP were used. Our results showed significant, positive associations between elevated coffee intake (ECI) (three or more cups of coffee per day) and NCP in verbal fluency, psychomotor speed (coding) and executive functioning. ECI might therefore preserve neurocognitive functioning in people living with HIV and HCV.

## 1. Introduction

Coffee is one of the most widely consumed drinks in the world, especially in high-resource settings [1]. It is associated with better overall health and a reduced risk of both mortality [2] and cancer [3] in the general population.

In people infected with hepatitis C virus (HCV), coffee consumption is associated with lower liver stiffness [4] and with decreased rates of liver disease progression and severity [5]. Specifically, an in vitro study showed that coffee extract and caffeic acid inhibit HCV viral propagation [6]. Elevated coffee intake (ECI) (three or more cups per day) is an independent predictor of improved virological response to peginterferon plus ribavirin therapy in patients with chronic HCV infection [7] and is associated with improved treatment tolerance.

In people living with HIV and HCV, previous research has shown that ECI can reduce the risk of mortality by 50% [8]. With regard to liver function, Yaya et al. pointed out that ECI is associated with a significantly reduced risk of advanced liver fibrosis in HIV-HCV coinfected patients, even in those with unhealthy alcohol use [9]. Furthermore, reduced levels of liver enzymes have been highlighted in patients with ECI by Morisco et al. [5] and Carrieri et al. [10]. Other beneficial effects of ECI in this population are its positive effects on insulin resistance [10], perceived toxicity and fatigue [11]. 

Apart from its beneficial effects on liver disease, coffee intake also significantly impacts cognition because of its stimulating effects on the central nervous system (CNS). In a study performed in 1875 healthy adults, habitual caffeine consumption was significantly related to better long-term memory performance and faster locomotor speed. No relationships were found between habitual caffeine consumption and short-term memory, information processing, planning and attention [12]. A meta-analysis showed a J-shaped association between coffee intake and incident cognitive disorders, with the lowest risk of incident cognitive disorders observed for a daily consumption level of 1–2 cups of coffee [13]. In the elderly, Haller et al. (2018) demonstrated an association between moderate caffeine consumption (from one to two cups of coffee/day) and better neurocognitive performance (NCP) and between moderate to ECI and better white matter preservation and cerebral blood flow [14]. ECI has also been associated with a reduced risk of Alzheimer’s disease [15]. Furthermore, people living with HIV and HCV experience an accelerated aging process [16] and suffer from neurocognitive aging.

Cognitive impairment is prevalent in HIV-HCV coinfected people, with rates ranging from 40% to 63% [17]. Compared with HIV mono-infected patients, coinfected patients have higher levels of cognitive impairment, particularly in information processing speed [18]. Vivithanaporn et al. showed that the presence of HCV coinfection in HIV-infected individuals is likely to increase the neurologic disease burden and risk of death [19]. With regard to the underlying mechanisms, an HCV-encoded protein, named Core, has been found to cause neuroinflammation and neuronal death by potentiating HIV-associated neurotoxicity [20].

No study, to date, has examined the association between coffee consumption and neurocognitive functioning in HIV-HCV coinfected patients. The present study aimed to analyse the relationship between coffee consumption and neurocognitive performance (NCP) in a sample of HIV-HCV coinfected patients, characterized by a high rate of HIV viral suppression.

## 2. Materials and Methods 

We used data from 139 HIV-HCV coinfected patients who participated in both the ANRS CO13 HEPAVIH cohort [21] and the HEPAVIH-Psy cross-sectional survey [22]. The latter was nested in the former and was designed to estimate the prevalence of mental health and substance use disorders in HIV-HCV coinfected patients recruited in 10 French HIV services between 2012 and 2014. Exclusion criteria for HEPAVIH-Psy were diagnosis of a current psychotic episode and neurological or medical disorders that may affect NCP, such as cerebrovascular disease or head trauma.

HEPAVIH-Psy provided data about current major depressive disorder (MDD) and NCP, the latter being assessed by measuring the following functions (with associated test/scale in brackets): visuospatial abilities and visual memory (Rey–Osterrieth complex figure test (ROCF) [23], vocabulary size or lexical access speed (verbal fluency task) [24], processing speed (coding task, a subtest of the fourth version of the Wechsler Adult Intelligence Scale (WAIS-IV)) [25] and executive functioning (Trail Making Test (TMT) part B minus A) [26]. The TMT B-A score was calculated as the difference between TMT-A and TMT-B times and is considered a measure of cognitive flexibility relatively independent of manual dexterity [27]. These cognitive functions, which have been shown to be sensitive enough to detect a possible neurocognitive impairment in HIV-infected individuals [28], were considered outcomes in our study. We tested coffee consumption in the previous six months and other factors, including age, gender, educational level, liver disease status (presence of cirrhosis), ongoing HCV treatment, HIV viral load and MDD, as potential correlates of neurocognitive performance. All of these variables were included in the HEPAVIH cohort and measured at the closest visit to the date of the HEPAVIH-Psy survey, except for MDD, which was documented in the HEPAVIH-Psy survey itself.

Our study outcomes were the five raw test scores measuring NCP (ROCF—direct copy and delayed reproduction, verbal fluency, coding, TMT B-A), with higher scores indicating better results for all tests/scales except TMT. Results for TMT are reported as the logarithm of the number of seconds required to complete the given task. Therefore, higher scores reflect greater impairment. Distributions of raw test scores are illustrated in Section 3.2. We used linear regression models to study the association between coffee intake during the previous six months (≥3 cups per day (ECI), ≤2 cups per day, no consumption) and each of the five outcomes. First, we selected all variables associated with the outcomes using a liberal *p*-value < 0.20 in the univariable analysis. We then built the five multivariable models. Only variables associated with at least one out of the five outcomes in univariable and multivariable analyses (using a *p*-value < 0.05) were included in order to have comparable multivariable models. Educational level was forced into all models, as it is an important cofactor of NCP.

## 3. Results

### 3.1. Study Population

Study patients were mostly men (66.9%), median (IQR) age was 50 (48–53) years, and 40.3% of patients had an educational level above or equal to the French high school diploma. A total of 91.3% of patients had an undetectable HIV viral load and 23.7% had cirrhosis. A total 28.8% reported ECI in the previous six months (Table 1).

### 3.2. Outcomes 

**Figure 1:**
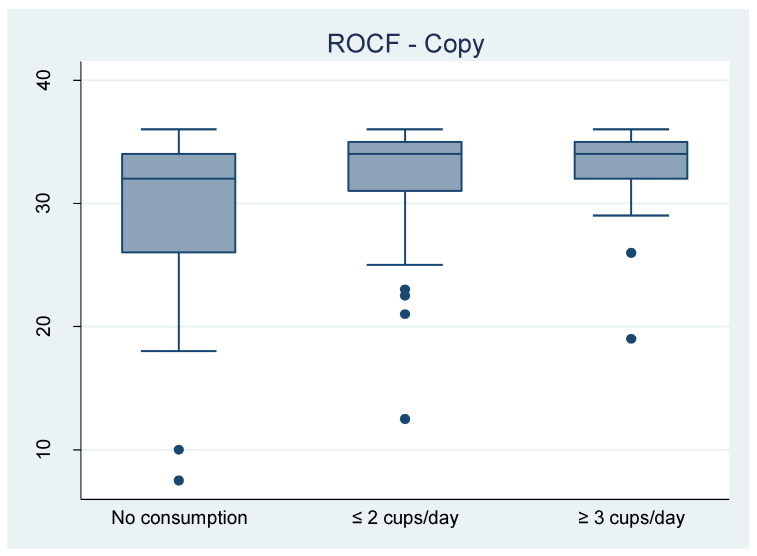

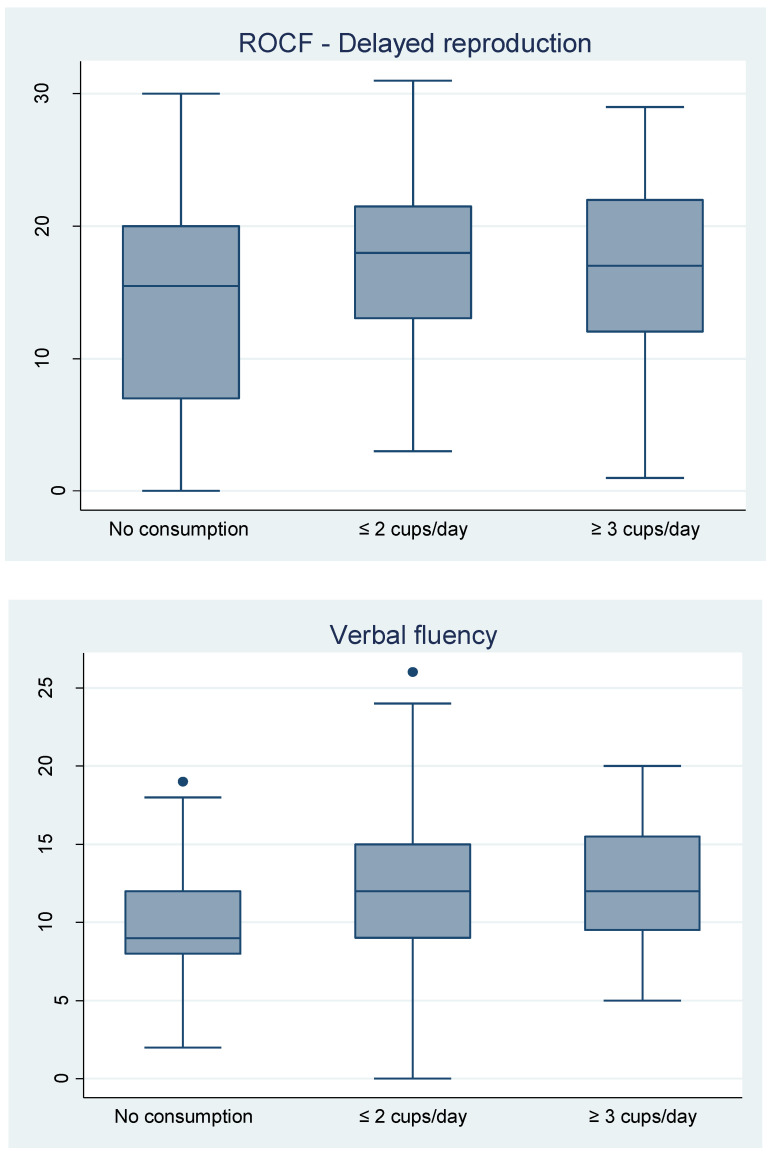

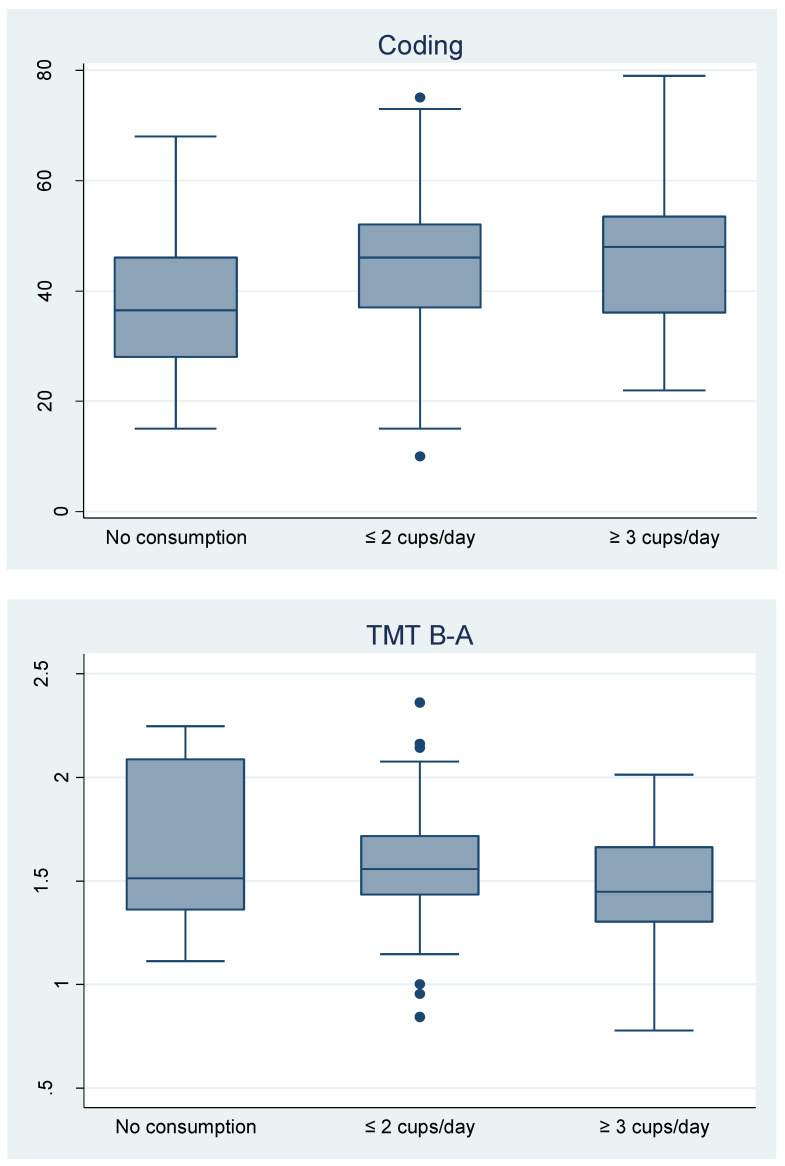
Distribution of raw test scores of HIV-HCV coinfected patients in the study population, the ANRS CO13 HEPAVIH cohort and the HEPAVIH-Psy cross-sectional survey (N = 139). Abbreviations: ROCF: Rey-Osterrieth complex figure; TMT B-A: Trail Making Test part B minus A.

The distributions of the five raw test scores measuring NCP (ROCF—direct copy and delayed reproduction, verbal fluency, coding, TMT B-A) are presented as boxplots. We stratified by coffee consumption, comparing the distributions of test scores in the three groups: no consumption, ≤ 2 cups/day and ≥ 3 cups/day (Figure 1).

### 3.3. Coffee Consumption Associated with Neurocognitive Performance in HIV-HCV Coinfected People

Interestingly, we found that ECI was positively associated with four of the five outcomes, as follows: ROCF (copy score only), verbal fluency, coding and TMT B-A. This result was confirmed after adjusting for clinical (presence of cirrhosis, ongoing HCV treatment, detectable HIV viral load, MDD), sociodemographic (age, gender, educational level) and socio-behavioural (cannabis use) correlates of the outcomes (Table 2).

## 4. Discussion

This is the first study to explore the relationship between coffee intake and neurocognitive performance in people coinfected with HIV and HCV. We showed that elevated coffee intake (ECI) (i.e., three cups or more per day) was associated with better NCP, as measured by the ROCF (direct copy only), verbal fluency, coding and TMT B-A tests. These results are clinically relevant given that the HIV-HCV coinfected population is doubly affected by their vulnerability to cognitive impairments and the burden of their diseases [17,19]. A meta-analysis comparing cognitive performance between HIV-HCV coinfected and HIV and HCV mono-infected patients showed significantly poorer information processing speed in the coinfected group [18]. Our results showed significant, positive associations between information processing speed (measured by the coding task test) and ECI. 

Our findings are in line with previous research in people living with HIV [29], showing the protective effects of moderate coffee intake on cognitive function. For example, Bragança and colleagues showed that regularly drinking espresso was associated with better Global Deficit Scores (GDS) and improved cognitive performance in five out of eight cognitive tests. They also found daily espresso consumption to be a positive predictor for performance in attention, working memory, executive functions and GDS. 

Interestingly, our results remained valid after adjustment for known correlates of neurocognitive impairment. We presume that this observed effect is not “acute” but attributable to prolonged exposure to ECI. 

In particular, the positive relationship between ECI and NCP persisted even after adjusting for known liver disease correlates (cirrhosis and ongoing HCV treatment), which suggests that the beneficial influence of coffee intake on NCP may occur irrespective of liver disease related factors. Accordingly, our results might be explained by a direct effect of caffeine on the CNS [30]. Caffeine targets specific brain regions involved in executive and verbal working memory functions [31], explaining the positive associations with NCP in verbal fluency observed in our study. In addition, caffeine enhances information processing speed and attention, which are two cognitive functions mobilised during the coding test [32]. 

With regard to the underlying mechanisms, our results may be explained, at least in part, by the antioxidant properties of coffee [33], which are able to counter the harmful effects of HCV- and HIV-induced neuro-inflammation. More specifically, chlorogenic acid, an important polyphenol found in coffee, has been shown to improve the oxidative system [34] and, therefore, may counter the inflammatory effects of HCV and HIV on the CNS. This is particularly relevant since HCV is characterized by high oxidative stress, which is shown to promote liver fibrosis, cirrhosis and cancer, as well as metabolic dysfunction [35]. This study’s strengths include rigorous control for several clinical (HIV viral load, presence of cirrhosis, treatment status) and socio-behavioural (age, gender, educational level) confounding factors. Moreover, MDD in the study population was diagnosed by psychiatrists and taken into account in our analysis.

Our study also has limitations. First, because it was cross-sectional, we were not able to infer causality for the associations found. Furthermore, we used raw scores as outcomes instead of a global deficit score, which is frequently used in other studies [18,29]. However, not aggregating our results into a single score enabled us to distinguish the cognitive functions assessed by the different tests and to provide more detailed results. Furthermore, we did not have information about the type of coffee consumed (caffeinated or decaffeinated, green or roasted), and we did not consider other caffeine sources, such as energy drinks, tea, chocolate or cocoa, which are likely to affect cognitive functioning [36]. Future research using consistent and comprehensive neuropsychological assessment batteries is needed in order to clarify the effects of coffee intake (including the cumulative effect of prolonged coffee consumption) on cognitive function and the mechanisms underlying these effects. It may also be useful to disentangle the effects of the numerous coffee compounds and their potential antioxidant activity on NCP and inflammation indicators in HIV-HCV coinfected patients to further explore these effects in certain categories of patients (such as patients with metabolic syndrome) and to assess the potential dose–response pattern of coffee intake on neurocognitive functioning in this population.

## 5. Conclusions

The strong relationship we found between coffee intake and NCP underlines the multiple benefits of coffee consumption in HIV-HCV coinfected people, ranging from reduced inflammation and risk of liver disease to reduced morbidity and mortality risk. Because cognitive deficits can have significant functional consequences for patients’ everyday lives—such as difficulties in remembering important information, reduced quality of life, and poor adherence to treatments—our results may have important implications for the planning of effective clinical management of these diseases. The effect of coffee and other functional food on HIV-HCV-related outcomes should also be included in the clinical and public health research agenda.

## Figures and Tables

**Table 1:** Characteristics of HIV-HCV coinfected patients in the study population, the ANRS CO13 HEPAVIH cohort and the HEPAVIH-Psy cross-sectional survey (N = 139).

	*N* (%)
Age, years	
Median (IQR)	50 (48–53)
Gender	
Male	93 (66.9)
Female	46 (33.1)
High school certificate *	
No	83 (59.7)
Yes	56 (40.3)
Current MDD (N = 137)	
No	107 (78.1)
Yes	30 (21.9)
HIV-related characteristics:	
CD4 count, cells/mm^3^ (N = 138)	
Median (IQR)	522 (346–726)
Detectable HIV viral load (N = 138)	
No	126 (91.3)
Yes	12 (8.7)
HCV-related characteristics:	
Ongoing HCV-treatment	
No	116 (83.5)
Yes	23 (16.6)
Presence of cirrhosis	
No	106 (76.3)
Yes	33 (23.7)
Cannabis use	
No	81 (58.3)
Yes	58 (41.7)
Coffee intake	
≥3 cups/day	40 (28.8)
≤2 cups/day	81 (58.3)
No consumption	18 (13.0)

* Educational level above or equal to the French Baccalaureate. Abbreviations: IQR—interquartile range; MDD—major depressive disorder; HIV—human immunodeficiency virus; HCV—hepatitis C virus.

**Table 2:** Factors associated with neurocognitive performance in HIV-HCV coinfected patients, multivariable linear regression models, the ANRS CO13 HEPAVIH cohort and the HEPAVIH-Psy cross-sectional survey (N = 139).

	ROCF				Verbal Fluency (*N* = 134)		Coding (*N* = 135)		TMT B-A ^1^ (*N* = 132)	
	Copy (*N* = 134)		Delayed Reproduction (*N* = 131) ^2^							
	Coefficient (95% CI)	*p*-Value	Coefficient (95% CI)	*p*-Value	Coefficient (95% CI)	*p*-Value	Coefficient (95% CI)	*p*-Value	Coefficient (95% CI)	*p*-Value
Coffee intake										
≤2 cups/day	3.35 (−0.28 to 6.99)	0.070	3.05 (−0.78 to 6.87)	0.117	2.32 (−0.33 to 4.97)	0.085	7.58 (0.18 to 14.97)	**0.045**	−0.11 (−0.28 to 0.07)	0.226
≥3 cups/day	4.63 (0.88 to 8.39)	**0.016**	2.70 (−1.44 to 6.84)	0.199	3.08 (0.18 to 5.97)	**0.037**	9.24 (1.26 to 17.36)	**0.024**	−0.27 (−0.47 to −0.07)	**0.009**
Age	0.10 (−0.04 to 0.25)	0.165	−0.09 (−0.26 to 0.09)	0.333	0.03 (−0.12 to 0.16)	0.701	−0.82 (−1.18 to −0.46)	**0.000**	0.00 (−0.01 to 0.01)	0.608
Educational level ^3^	−1.19 (−2.79 to 0.41)	0.145	2.06 (−0.08 to 4.20)	0.059	0.59 (−1.25 to 2.42)	0.527	1.03 (−2.93 to 5.00)	0.607	−0.06 (−0.17 to 0.05)	0.302
Current MDD	−1.88 (−4.61 to 0.85)	0.176	−2.81 (−5.23 to −0.38)	**0.024**	−0.73 (−2.74 to 1.29)	0.477	−6.75 (−11.50 to −2.00)	**0.006**	0.05 ( −0.11 to 0.22)	0.529
Presence of cirrhosis	−2.28 (−4.49 to −0.07)	**0.043**	−1.12 (−3.60 to 1.38)	0.380	−1.07 (−3.04 to 0.89)	0.282	−4.38 (−9.23 to 0.47)	0.076	0.07 (−0.06 to 0.19)	0.288
Ongoing HCV treatment	0.26 (−1.67 to 2.18)	0.792	0.13 (−3.15 to 3.40)	0.939	−2.43 (−4.72 to −0.13)	**0.039**	−9.01 (−14.14 to −3.88)	**0.001**	0.00 (−0.14 to 0.15)	0.988
Detectable HIV viral load	0.13 (−1.83 to 2.09)	0.896	−0.71 (−3.56 to 2.14)	0.623	−1.73 (−4.41 to 0.96)	0.205	−10.97 (−17.38 to −4.55)	**0.001**	0.21 (0.05 to 0.38)	**0.013**
Cannabis use	−0.89 (−2.65 to 0.87)	0.320	−0.46 (−2.70 to 1.78)	0.685	−1.77 (−3.61 to 0.06)	0.058	−4.91 (−8.98 to −0.84)	**0.019**	0.09 (−0.03 to 0.20)	0.149

^1^ TMT B-A was log10-transformed so results must be interpreted as 10^Est. ^2^ Adjusted for quality of the copy and for reproduction time (log10 transformed). ^3^ Educational level above or equal to the French Baccalaureate. Abbreviations: ROCF: Rey-Osterrieth complex figure; MDD: major depressive disorder; HCV: hepatitis C virus; TMT B-A: trail making test part B minus A.

## Appendix A

The ANRS CO13 HEPAVIH Study Group: *Scientific Committee:* D.Salmon (co-Principal investigator), L.Wittkop (co-Principal Investigator & Methodologist), P.Sogni (co-Principal Investigator), L. Esterle (project manager), P.Trimoulet, J.Izopet, L.Serfaty, V.Paradis, B.Spire, P.Carrieri, M.A.Valantin, G.Pialoux, J.Chas, I.Poizot-Martin, K.Barange, A.Naqvi, E.Rosenthal, A.Bicart-See, O.Bouchaud, A.Gervais, C.Lascoux-Combe, C.Goujard, K.Lacombe, C.Duvivier, D.Neau, P.Morlat, F.Bani-Sadr, L.Meyer, F.Boufassa, B.Autran, A.M.Roque, C.Solas, H.Fontaine, D.Costagliola, L.Piroth, A.Simon, D.Zucman, F.Boué, P.Miailhes, E.Billaud, H.Aumaître, D.Rey, G.Peytavin, V.Petrov-Sanchez, D.Lebrasseur-Longuet. *Clinical Centres* (ward/participating physicians): APHP, Hôpitaux Universitaires Paris Centre, Paris (Médecine Interne et Maladies Infectieuses: D. Salmon, R.Usubillaga; Hépato-gastro-entérologie: P.Sogni; Anatomo-pathologie: B.Terris; Virologie: P.Tremeaux); APHP Pitié-Salpétrière, Paris (Maladies Infectieuses et Tropicales: C.Katlama, M.A.Valantin, H.Stitou; Médecine Interne: A.Simon, P.Cacoub, S.Nafissa; Hépato-gastro-entérologie: Y.Benhamou; Anatomo-pathologie: F.Charlotte; Virologie: S.Fourati); APHM Sainte-Marguerite, Marseille (Service d’Immuno-Hématologie Clinique: I.Poizot-Martin, O.Zaegel, H.Laroche; Virologie: C.Tamalet); APHP Tenon, Paris (Maladies Infectieuses et Tropicales: G.Pialoux, J.Chas; Anatomo-pathologie: P.Callard, F.Bendjaballah; Virologie: C.Amiel, C.Le Pendeven); CHU Purpan, Toulouse (Maladies Infectieuses et Tropicales: B. Marchou; Médecine interne: L.Alric; Hépato-gastro-entérologie: K.Barange, S.Metivier; Anatomo-pathologie: J.Selves; Virologie: F.Larroquette); CHU Archet, Nice (Médecine Interne: E.Rosenthal; Infectiologie: A.Naqvi, V.Rio; Anatomo-pathologie: J.Haudebourg, M.C.Saint-Paul; Virologie: A. De Monte, V.Giordanengo, C.Partouche); APHP Avicenne, Bobigny (Médecine Interne – Unité VIH: O.Bouchaud; Anatomo-pathologie: A.Martin, M.Ziol; Virologie: Y.Baazia, V.Iwaka-Bande, A.Gerber); Hôpital Joseph Ducuing, Toulouse (Médecine Interne: M.Uzan, A.Bicart-See, D.Garipuy, M.J.Ferro-Collados; Anatomo-pathologie: J.Selves; Virologie: F.Nicot); APHP Bichat – Claude-Bernard, Paris (Maladies Infectieuses: A.Gervais, Y.Yazdanpanah; Anatomo-pathologie: H.Adle-Biassette; Virologie: G.Alexandre, Pharmacologie: G.Peytavin); APHP Saint-Louis, Paris (Maladies infectieuses: C.Lascoux-Combe, J.M.Molina; Anatomo-pathologie: P.Bertheau; Virologie: M.L.Chaix, C. Delaugerre, S. Maylin); APHP Saint-Antoine (Maladies Infectieuses et Tropicales: K. Lacombe, J. Bottero; J. Krause, P.M. Girard, Anatomo-pathologie: D. Wendum, P. Cervera, J. Adam; Virologie: C. Viala); APHP, Hôpitaux Paris Sud, Bicêtre, Paris (Maladies Infectieuses et Tropicales: D. Vittecocq; Médecine Interne: C. Goujard, Y. Quertainmont, E. Teicher; Virologie: C. Pallier); APHP Necker, Paris (Maladies Infectieuses et Tropicales: O. Lortholary, C. Duvivier, C. Rouzaud, J. Lourenco, F. Touam, C. Louisin: Virologie: V. Avettand-Fenoel, E. Gardiennet, A. Mélard); CHU Bordeaux Hôpital Pellegrin, Bordeaux (Maladies Infectieuses et Tropicales: D. Neau, A. Ochoa, E. Blanchard, S. Castet-Lafarie, C. Cazanave, D. Malvy, M. Dupon, H. Dutronc, F. Dauchy, L. Lacaze-Buzy, A. Desclaux; Anatomo-pathologie: P. Bioulac-Sage; Virologie: P. Trimoulet, S. Reigadas; CHU Bordeaux Hôpital Saint-André, Bordeaux (Médecine Interne et Maladies Infectieuses: P. Morlat, D. Lacoste, F. Bonnet, N. Bernard, M. Hessamfar, J, F. Paccalin, C. Martell, M. C. Pertusa, M. Vandenhende, P. Mercié, D. Malvy, T. Pistone, M.C. Receveur, M. Méchain, P. Duffau, C Rivoisy, I. Faure, S. Caldato; Anatomo-pathologie: P. Bioulac-Sage; Virologie: P. Trimoulet, S. Reigadas, P. Bellecave, C. Tumiotto); CHU Bordeaux Hôpital du Haut-Levêque, Bordeaux (Médecine Interne: J.L. Pellegrin, J.F. Viallard, E. Lazzaro, C. Greib; Anatomo-pathologie: P. Bioulac-Sage; Virologie: P. Trimoulet, S. Reigadas); Hôpital FOCH, Suresnes (Médecine Interne: D. Zucman, C. Majerholc; Virologie: M. Brollo, E. Farfour); APHP Antoine Béclère, Clamart (Médecine Interne: F. Boué, J. Polo Devoto, I. Kansau, V. Chambrin, C. Pignon, L. Berroukeche, R. Fior, V. Martinez, S. Abgrall, M. Favier; Virologie: C. Deback); CHU Henri Mondor, Créteil (Immunologie Clinique: Y. Lévy, S. Dominguez, J.D. Lelièvre, A.S. Lascaux, G. Melica); CHU Nantes Hôpital Hôtel Dieu, Nantes (Maladies Infectieuses et Tropicales: E. Billaud, F. Raffi, C. Allavena, V. Reliquet, D. Boutoille, C. Biron; M. Lefebvre, N. Hall, S. Bouchez; Virologie: A. Rodallec, L. Le Guen, C. Hemon); Hôpital de la Croix Rousse, Lyon (Maladies Infectieuses et Tropicales: P. Miailhes, D. Peyramond, C. Chidiac, F. Ader, F. Biron, A. Boibieux, L. Cotte, T. Ferry, T. Perpoint, J. Koffi, F. Zoulim, F. Bailly, P. Lack, M. Maynard, S. Radenne, M. Amiri, F Valour; Hépato-gastro-entérologie: J. Koffi, F. Zoulim, F. Bailly, P. Lack, M. Maynard, S. Radenne, C. Augustin-Normand; Virologie: C. Scholtes, T.T. Le-Thi); CHU Dijon, Dijon (Département d’infectiologie: L. Piroth, P. Chavanet M. Duong Van Huyen, M. Buisson, A. Waldner-Combernoux, S. Mahy, A. Salmon Rousseau, C. Martins); CH Perpignan, Perpignan (Maladies infectieuses et tropicales: H. Aumaître, Virologie: S. Galim); CHU Robert Debré, Reims (Médecine interne, maladies infectieuses et immunologie clinique: F. Bani-Sadr, D. Lambert, Y Nguyen, J.L. Berger, M. Hentzien, Virologie: V. Brodard); CHRU Strasbourg (Le Trait d’Union: D Rey, M Partisani, ML Batard, C Cheneau, M Priester, C Bernard-Henry, E de Mautort, P Fischer, Virologie: P Gantner et S Fafi-Kremer). *Data collection*: F.Roustant, P. Platterier, I. Kmiec, L. Traore, S. Lepuil, S. Parlier, V. Sicart-Payssan, E. Bedel, S. Anriamiandrisoa, C. Pomes, F. Touam, C. Louisin, M. Mole, C. Bolliot, P Catalan, M. Mebarki, A. Adda-Lievin, P. Thilbaut, Y. Ousidhoum, F.Z. Makhoukhi, O. Braik, R. Bayoud, C. Gatey, M.P. Pietri, V. Le Baut, R. Ben Rayana, D. Bornarel, C. Chesnel, D. Beniken, M. Pauchard, S. Akel, S. Caldato, C. Lions, A. Ivanova, A-S. Ritleg, C. Debreux, L. Chalal, J.Zelie, H. Hue, A. Soria, M. Cavellec, S. Breau, A. Joulie, P. Fisher, S. Gohier, D. Croisier-Bertin, S. Ogoudjobi, C. Brochier, V. Thoirain-Galvan, M. Le Cam. *Management, statistical analyses:* P. Carrieri, M. Chalouni, V. Conte, L. Dequae-Merchadou, M. Desvallees, L. Esterle, C. Gilbert, S. Gillet, R. Knight, T. Lemboub, F. Marcellin, L. Michel, M. Mora, C. Protopopescu, P. Roux, B. Spire, S. Tezkratt, T. Barré, M. Baudoin, M. Santos, V. Di Beo, M.Nishimwe, L Wittkop.

HEPAVIH-Psy clinical centres (ward): Centre hospitalo-universitaire (CHU) Cochin (Médecine Interne et Maladies Infectieuses); CHU Pitié-Salpêtrière (Maladies Infectieuses et Tropicales); CHU Sainte-Marguerite, Marseille (Service d’Immuno-Hématologie Clinique/CISIH); CHU Purpan Toulouse (Maladies Infectieuses et Tropicales); CHU Archet, Nice (Médecine Interne); CHU Saint-Louis (Médecine Interne); CHU Saint Antoine (Maladies Infectieuses et Tropicales); CHU Necker (Maladies Infectieuses et Tropicales); ANRS CO3 Aquitaine cohort (Hôpital Saint André and Hôpital Pellegrin).
